# Bis(μ-2-{[2-(1,3-benzothia­zol-2-yl)hydrazinyl­idene]meth­yl}-6-meth­oxy­phenolato)bis­[dinitratodysprosium(III)] methanol disolvate

**DOI:** 10.1107/S1600536811019118

**Published:** 2011-05-25

**Authors:** Xuebin Xu, Shuai Ding, Si Shen, Jinkui Tang, Zhiliang Liu

**Affiliations:** a College of Chemistry and Chemical Engineering, Inner Mongolia University, Hohhot 010021, People’s Republic of China; b State Key Laboratory of Rare Earth Resource Utilization, Changchun Institute of Applied Chemistry, Chinese Academy of Sciences, Changchun 130022, People’s Republic of China.

## Abstract

In the centrosymmetric dinuclear title compound, [Dy_2_(C_15_H_12_N_3_O_2_S)_2_(NO_3_)_4_]·2CH_3_OH, the two Dy^III^ atoms are coordinated by two deprotonated 2-{[2-(1,3-benzothia­zol-2-yl)hydrazinyl­idene]meth­yl}-6-meth­oxy­phenol ligands and four nitrate ions, all of which are chelating. The crystal packing is stabilized by inter­molecular N—H⋯O hydrogen bonds and weak O—H⋯O inter­actions, forming a two-dimensional network parallel to (010).

## Related literature

For applications of dysprosium complexes in data storage and processing, see: Lin *et al.* (2010 ▶). For the preparation of the 2-{[2-(1,3-benzothia­zol-2-yl)hydrazinyl­idene]meth­yl}-6-meth­oxy­phenol ligand, see: Patil *et al.* (2009 ▶). For related structures, see: Lin & Hong (2009 ▶); Lin *et al.* (2008 ▶); Xu *et al.* (2010 ▶).
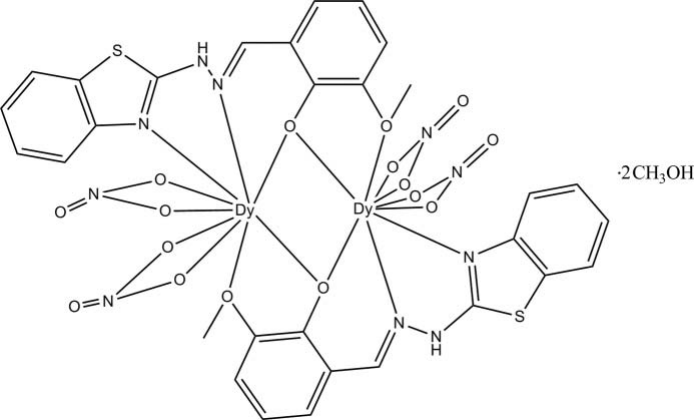


## Experimental

### 

#### Crystal data

[Dy_2_(C_15_H_12_N_3_O_2_S)_2_(NO_3_)_4_]·2CH_4_O
*M* *_r_* = 1233.82
Triclinic, 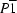

*a* = 9.6191 (6) Å
*b* = 10.1002 (7) Å
*c* = 11.6151 (8) Å
α = 112.045 (1)°
β = 105.065 (1)°
γ = 93.154 (1)°
*V* = 995.23 (12) Å^3^
*Z* = 1
Mo *K*α radiation
μ = 3.92 mm^−1^
*T* = 159 K
0.20 × 0.10 × 0.10 mm

#### Data collection

Rigaku Saturn CCD area-detector diffractometer
Absorption correction: multi-scan (*CrystalClear*; Rigaku, 2002 ▶) *T*_min_ = 0.631, *T*_max_ = 0.676
5083 measured reflections
3502 independent reflections
3162 reflections with *I* > 2σ(*I*)
*R*_int_ = 0.016

#### Refinement

*R*[*F* ^2^ > 2σ(*F* ^2^)] = 0.025
*wR*(*F* ^2^) = 0.057
*S* = 1.07
3502 reflections
292 parameters
H-atom parameters constrained
Δρ_max_ = 0.83 e Å^−3^
Δρ_min_ = −0.51 e Å^−3^

### 

Data collection: *CrystalClear* (Rigaku, 2002 ▶); cell refinement: *CrystalClear*; data reduction: *CrystalClear*; program(s) used to solve structure: *SHELXS97* (Sheldrick, 2008 ▶); program(s) used to refine structure: *SHELXL97* (Sheldrick, 2008 ▶); molecular graphics: *DIAMOND* (Brandenburg & Putz, 2006 ▶) and *XP* (Siemens, 1994 ▶); software used to prepare material for publication: *publCIF* (Westrip, 2010 ▶).

## Supplementary Material

Crystal structure: contains datablocks I, global. DOI: 10.1107/S1600536811019118/jj2083sup1.cif

Structure factors: contains datablocks I. DOI: 10.1107/S1600536811019118/jj2083Isup2.hkl

Additional supplementary materials:  crystallographic information; 3D view; checkCIF report

## Figures and Tables

**Table 1:** Selected bond lengths (Å)

Dy1—O2	2.280 (3)
Dy1—O2^i^	2.374 (2)
Dy1—O1^i^	2.394 (3)
Dy1—O4	2.422 (3)
Dy1—O3	2.433 (3)
Dy1—N4	2.461 (3)
Dy1—O6	2.471 (3)
Dy1—N5	2.494 (3)
Dy1—O8	2.530 (3)

Symmetry code: (i) 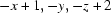.

**Table 2:** Hydrogen-bond geometry (Å, °)

*D*—H⋯*A*	*D*—H	H⋯*A*	*D*⋯*A*	*D*—H⋯*A*
O9—H9*A*⋯O3^ii^	0.84	2.64	3.206 (4)	126
O9—H9*A*⋯O8^iii^	0.84	2.24	3.049 (4)	161
N9—H9⋯O9^iv^	0.88	1.91	2.751 (4)	160

Symmetry codes: (ii) 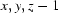; (iii) 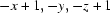; (iv) 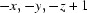.

**Fig. 1.**
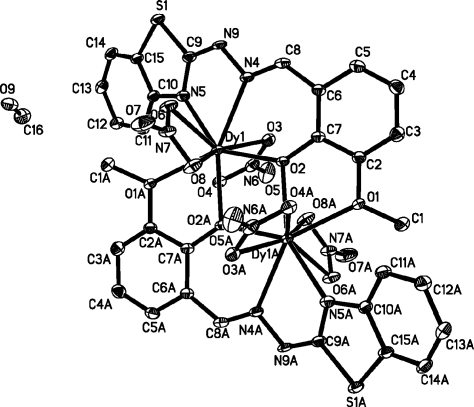
The molecular structure of the title compound, with atom labels and 30% probability displacement ellipsoids. Hydrogen atoms are omitted for clarity.

**Fig. 2.**
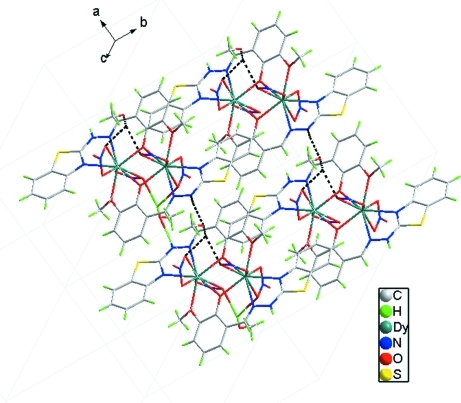
Packing diagram of the title compound. Hydrogen bonds are shown as dashed lines.

## supplementary crystallographic information

### Comment

Dysprosium complexes continue to attract significant attention because of their potential applications in data storage and processing (Lin *et al.* 2010). In order to explore the relationship between these applications and their structures, a series of dinuclear dysprosium coordination compounds have been structural characterized (Lin *et al.* 2008; Xu *et al.* 2010). In support of our continued research in this area, we report here a new dinuclear dysprosium complex, [Dy_2_(C_15_H_12_N_3_O_2_S)_2_(NO_3_)_4_].CH_3_OH.

In the title compound the asymmetric unit consists of one Dy^III^ ion, one deprotonated 2-[(benzothiazol-2-yl)hydrazonomethyl]-6-methoxyphenol ligand and two nitrate ions (Fig. 1). The centrosymmetric dinuclear complex is composed of two nine-coordinate Dy^III^ ions bridged by phenoxo groups (O2, O2a) from the ligands with a Dy1—O2—Dy1a angle of 106.041 (100) ° and a Dy···Dy distance equal to 3.7184 (3) Å.  The central core Dy_2_O_2_ appears to be nearly rhombic with the two Dy—O2 distances being 2.37 Å and 2.28 Å, respectively.  Crystal packing is stabiized by N—H···O hydrogen bonds and weak O—H···O intermolecular interactions forming a two-dimensional network (Fig. 2).

### Experimental

A mixture of Dy(NO_3_)_3_.6H_2_O (0.1 mmol), 2-hydrazino benzothiazole (0.1 mmol), 2-hydroxy-3-methoxy-5-((4-methoxyphenyl)diazenyl)benzaldehyde (0.1 mmol), methanol (10 ml), Et_3_N (0.3 mmol) was sealed in a glass vessel (20 ml, capacity) and the solution was heated at 363 K for 1 h under autogenous pressure. After the mixture was allowed to cool to room temperature, yellow block single crystals were isolated from the vessel.

### Refinement

The H atoms were placed in geometrically idealized positions (C—H = 0.95 Å and O—H = 0.82–0.84 Å), with *U*_iso_(H) = 1.2Ueq(C) and *U*_iso_(H) = 1.5Ueq(O).

### Crystal data

**Table:**

[Dy_2_(C_15_H_12_N_3_O_2_S)_2_(NO_3_)_4_]·2CH_4_O	*Z* = 1
*M**_r_* = 1233.82	*F*(000) = 602
Triclinic, *P*1	*D*_x_ = 2.059 Mg m^−^^3^
Hall symbol: -P 1	Mo *K*α radiation, λ = 0.71073 Å
*a* = 9.6191 (6) Å	Cell parameters from 3776 reflections
*b* = 10.1002 (7) Å	θ = 2.3–25.1°
*c* = 11.6151 (8) Å	µ = 3.92 mm^−^^1^
α = 112.045 (1)°	*T* = 159 K
β = 105.065 (1)°	Block, yellow
γ = 93.154 (1)°	0.20 × 0.10 × 0.10 mm
*V* = 995.23 (12) Å^3^	

### Data collection

**Table:**

Rigaku Saturn CCD area-detector diffractometer	3502 independent reflections
Radiation source: rotating anode	3162 reflections with *I* > 2σ(*I*)
confocal	*R*_int_ = 0.016
Detector resolution: 7.31 pixels mm^-1^	θ_max_ = 25.1°, θ_min_ = 2.0°
ω and φ scans	*h* = −11→11
Absorption correction: multi-scan (*CrystalClear*; Rigaku, 2002)	*k* = −12→7
*T*_min_ = 0.631, *T*_max_ = 0.676	*l* = −13→13
5083 measured reflections	

### Refinement

**Table:**

Refinement on *F*^2^	Primary atom site location: structure-invariant direct methods
Least-squares matrix: full	Secondary atom site location: difference Fourier map
*R*[*F*^2^ > 2σ(*F*^2^)] = 0.025	Hydrogen site location: inferred from neighbouring sites
*wR*(*F*^2^) = 0.057	H-atom parameters constrained
*S* = 1.07	*w* = 1/[σ^2^(*F*_o_^2^) + (0.0289*P*)^2^ + 0.127*P*] where *P* = (*F*_o_^2^ + 2*F*_c_^2^)/3
3502 reflections	(Δ/σ)_max_ = 0.001
292 parameters	Δρ_max_ = 0.83 e Å^−^^3^
0 restraints	Δρ_min_ = −0.51 e Å^−^^3^

### Special details

**Table:**

Geometry. All e.s.d.'s (except the e.s.d. in the dihedral angle between two l.s. planes) are estimated using the full covariance matrix. The cell e.s.d.'s are taken into account individually in the estimation of e.s.d.'s in distances, angles and torsion angles; correlations between e.s.d.'s in cell parameters are only used when they are defined by crystal symmetry. An approximate (isotropic) treatment of cell e.s.d.'s is used for estimating e.s.d.'s involving l.s. planes.
Refinement. Refinement of *F*^2^ against ALL reflections. The weighted *R*-factor *wR* and goodness of fit *S* are based on *F*^2^, conventional *R*-factors *R* are based on *F*, with *F* set to zero for negative *F*^2^. The threshold expression of *F*^2^ > σ(*F*^2^) is used only for calculating *R*-factors(gt) *etc*. and is not relevant to the choice of reflections for refinement. *R*-factors based on *F*^2^ are statistically about twice as large as those based on *F*, and *R*- factors based on ALL data will be even larger.

### Fractional atomic coordinates and isotropic or equivalent isotropic displacement parameters (Å^2^)

**Table:**

	*x*	*y*	*z*	*U*_iso_*/*U*_eq_	
C1	0.5488 (5)	−0.1481 (6)	1.3423 (4)	0.0419 (12)	
H1A	0.4829	−0.0989	1.3895	0.063*	
H1B	0.6488	−0.0946	1.3876	0.063*	
H1C	0.5453	−0.2468	1.3387	0.063*	
C2	0.3550 (4)	−0.2085 (4)	1.1420 (4)	0.0262 (9)	
C3	0.2744 (5)	−0.3007 (5)	1.1704 (4)	0.0328 (10)	
H3	0.3194	−0.3323	1.2363	0.039*	
C4	0.1277 (5)	−0.3481 (5)	1.1034 (4)	0.0349 (10)	
H4	0.0711	−0.4143	1.1208	0.042*	
C5	0.0654 (4)	−0.2974 (5)	1.0113 (4)	0.0314 (10)	
H5	−0.0365	−0.3263	0.9677	0.038*	
C6	0.1455 (4)	−0.2054 (4)	0.9789 (4)	0.0259 (9)	
C7	0.2972 (4)	−0.1615 (4)	1.0421 (4)	0.0237 (8)	
C8	0.0613 (4)	−0.1613 (5)	0.8805 (4)	0.0308 (10)	
H8	−0.0395	−0.2025	0.8423	0.037*	
C9	0.0364 (4)	0.0701 (5)	0.7244 (4)	0.0285 (9)	
C10	0.1564 (4)	0.2767 (4)	0.7467 (4)	0.0265 (9)	
C11	0.2636 (4)	0.3998 (5)	0.7988 (4)	0.0309 (10)	
H11	0.3530	0.4075	0.8617	0.037*	
C12	0.2378 (5)	0.5099 (5)	0.7573 (4)	0.0335 (10)	
H12	0.3112	0.5930	0.7904	0.040*	
C13	0.1059 (5)	0.5011 (5)	0.6680 (4)	0.0354 (10)	
H13	0.0902	0.5791	0.6421	0.042*	
C14	−0.0026 (5)	0.3818 (5)	0.6163 (4)	0.0335 (10)	
H14	−0.0930	0.3764	0.5557	0.040*	
C15	0.0251 (4)	0.2701 (5)	0.6558 (4)	0.0284 (9)	
C16	0.2951 (6)	0.3287 (6)	0.4354 (5)	0.0540 (14)	
H16A	0.2585	0.3862	0.3858	0.081*	
H16C	0.3978	0.3682	0.4862	0.081*	
H16B	0.2374	0.3326	0.4945	0.081*	
Dy1	0.358298 (18)	0.05304 (2)	0.890365 (17)	0.02346 (8)	
N4	0.1097 (3)	−0.0726 (4)	0.8405 (3)	0.0283 (8)	
N5	0.1632 (3)	0.1587 (4)	0.7829 (3)	0.0274 (8)	
N6	0.3542 (4)	0.3093 (4)	1.1063 (3)	0.0345 (9)	
N7	0.3601 (4)	−0.1898 (4)	0.6517 (3)	0.0327 (8)	
N9	0.0038 (3)	−0.0478 (4)	0.7456 (3)	0.0327 (8)	
H9	−0.0802	−0.1070	0.7020	0.039*	
O1	0.5034 (3)	−0.1543 (3)	1.2102 (3)	0.0291 (6)	
O2	0.3863 (3)	−0.0823 (3)	1.0113 (2)	0.0249 (6)	
O3	0.2717 (3)	0.1897 (3)	1.0724 (3)	0.0340 (7)	
O4	0.4335 (3)	0.3079 (3)	1.0330 (3)	0.0337 (7)	
O5	0.3585 (4)	0.4152 (4)	1.2012 (3)	0.0574 (10)	
O6	0.2613 (3)	−0.1127 (3)	0.6575 (3)	0.0371 (7)	
O7	0.3620 (4)	−0.2850 (4)	0.5512 (3)	0.0580 (10)	
O8	0.4579 (3)	−0.1600 (3)	0.7589 (3)	0.0416 (8)	
O9	0.2828 (3)	0.1836 (4)	0.3486 (3)	0.0452 (8)	
H9A	0.3398	0.1790	0.3038	0.068*	
S1	−0.09430 (11)	0.11034 (12)	0.61321 (10)	0.0315 (2)	

### Atomic displacement parameters (Å^2^)

**Table:**

	*U*^11^	*U*^22^	*U*^33^	*U*^12^	*U*^13^	*U*^23^
C1	0.033 (2)	0.062 (3)	0.028 (2)	0.002 (2)	0.0030 (19)	0.020 (2)
C2	0.021 (2)	0.029 (2)	0.027 (2)	0.0045 (17)	0.0058 (17)	0.0110 (18)
C3	0.036 (2)	0.029 (2)	0.033 (2)	0.006 (2)	0.0075 (19)	0.014 (2)
C4	0.033 (2)	0.033 (2)	0.041 (3)	0.002 (2)	0.011 (2)	0.017 (2)
C5	0.022 (2)	0.035 (2)	0.034 (2)	0.0027 (18)	0.0075 (18)	0.011 (2)
C6	0.0188 (19)	0.028 (2)	0.030 (2)	0.0037 (17)	0.0051 (17)	0.0118 (18)
C7	0.0183 (19)	0.022 (2)	0.028 (2)	0.0031 (16)	0.0038 (16)	0.0095 (18)
C8	0.016 (2)	0.037 (3)	0.033 (2)	0.0030 (18)	0.0006 (17)	0.013 (2)
C9	0.019 (2)	0.037 (2)	0.028 (2)	0.0086 (18)	0.0036 (17)	0.013 (2)
C10	0.023 (2)	0.032 (2)	0.024 (2)	0.0070 (18)	0.0049 (17)	0.0117 (18)
C11	0.022 (2)	0.039 (3)	0.030 (2)	0.0060 (19)	0.0039 (17)	0.014 (2)
C12	0.029 (2)	0.035 (2)	0.032 (2)	0.003 (2)	0.0046 (19)	0.012 (2)
C13	0.039 (2)	0.039 (3)	0.031 (2)	0.011 (2)	0.006 (2)	0.019 (2)
C14	0.031 (2)	0.042 (3)	0.028 (2)	0.012 (2)	0.0021 (19)	0.019 (2)
C15	0.020 (2)	0.038 (2)	0.022 (2)	0.0059 (18)	0.0028 (16)	0.0086 (19)
C16	0.043 (3)	0.069 (4)	0.044 (3)	0.009 (3)	0.011 (2)	0.018 (3)
Dy1	0.01390 (10)	0.03118 (12)	0.02375 (11)	0.00321 (8)	0.00021 (7)	0.01318 (9)
N4	0.0175 (16)	0.033 (2)	0.0301 (18)	−0.0005 (15)	−0.0019 (14)	0.0156 (16)
N5	0.0207 (17)	0.0310 (19)	0.0281 (18)	0.0054 (15)	0.0046 (14)	0.0110 (16)
N6	0.033 (2)	0.039 (2)	0.029 (2)	0.0144 (18)	0.0035 (17)	0.0152 (19)
N7	0.0199 (18)	0.036 (2)	0.031 (2)	0.0019 (16)	−0.0010 (15)	0.0067 (18)
N9	0.0144 (16)	0.045 (2)	0.0347 (19)	−0.0018 (16)	−0.0072 (14)	0.0229 (18)
O1	0.0195 (13)	0.0415 (17)	0.0277 (15)	0.0034 (13)	0.0010 (11)	0.0199 (14)
O2	0.0146 (13)	0.0316 (16)	0.0279 (14)	0.0016 (12)	0.0007 (11)	0.0156 (13)
O3	0.0291 (16)	0.0430 (19)	0.0312 (16)	0.0091 (15)	0.0063 (13)	0.0177 (15)
O4	0.0285 (15)	0.0393 (18)	0.0298 (15)	0.0044 (14)	0.0036 (13)	0.0138 (14)
O5	0.086 (3)	0.041 (2)	0.041 (2)	0.021 (2)	0.0250 (19)	0.0073 (18)
O6	0.0229 (15)	0.0449 (19)	0.0318 (16)	0.0119 (14)	−0.0016 (13)	0.0087 (15)
O7	0.0373 (19)	0.066 (3)	0.037 (2)	0.0132 (18)	−0.0008 (16)	−0.0066 (19)
O8	0.0351 (17)	0.0463 (19)	0.0321 (16)	0.0152 (15)	−0.0033 (14)	0.0117 (15)
O9	0.0258 (17)	0.057 (2)	0.046 (2)	−0.0010 (16)	0.0079 (14)	0.0163 (18)
S1	0.0212 (5)	0.0388 (6)	0.0302 (5)	0.0037 (5)	−0.0020 (4)	0.0161 (5)

### Geometric parameters (Å, °)

**Table:**

C1—O1	1.459 (5)	C13—H13	0.9500
C1—H1A	0.9800	C14—C15	1.384 (6)
C1—H1B	0.9800	C14—H14	0.9500
C1—H1C	0.9800	C15—S1	1.752 (4)
C2—C3	1.364 (6)	C16—O9	1.410 (6)
C2—O1	1.402 (4)	C16—H16A	0.9800
C2—C7	1.410 (5)	C16—H16C	0.9800
C3—C4	1.379 (6)	C16—H16B	0.9800
C3—H3	0.9500	Dy1—O2	2.280 (3)
C4—C5	1.370 (6)	Dy1—O2^i^	2.374 (2)
C4—H4	0.9500	Dy1—O1^i^	2.394 (3)
C5—C6	1.390 (5)	Dy1—O4	2.422 (3)
C5—H5	0.9500	Dy1—O3	2.433 (3)
C6—C7	1.412 (5)	Dy1—N4	2.461 (3)
C6—C8	1.451 (5)	Dy1—O6	2.471 (3)
C7—O2	1.342 (4)	Dy1—N5	2.494 (3)
C8—N4	1.270 (5)	Dy1—O8	2.530 (3)
C8—H8	0.9500	Dy1—Dy1^i^	3.7184 (4)
C9—N5	1.320 (5)	N4—N9	1.402 (4)
C9—N9	1.340 (5)	N6—O5	1.203 (5)
C9—S1	1.738 (4)	N6—O3	1.273 (5)
C10—C11	1.395 (6)	N6—O4	1.279 (4)
C10—C15	1.399 (5)	N7—O7	1.208 (5)
C10—N5	1.405 (5)	N7—O6	1.261 (4)
C11—C12	1.379 (6)	N7—O8	1.266 (4)
C11—H11	0.9500	N9—H9	0.8800
C12—C13	1.388 (6)	O1—Dy1^i^	2.394 (3)
C12—H12	0.9500	O2—Dy1^i^	2.374 (2)
C13—C14	1.376 (6)	O9—H9A	0.8400
			
O1—C1—H1A	109.5	O4—Dy1—O3	52.63 (10)
O1—C1—H1B	109.5	O2—Dy1—N4	75.53 (10)
H1A—C1—H1B	109.5	O2^i^—Dy1—N4	149.07 (10)
O1—C1—H1C	109.5	O1^i^—Dy1—N4	138.80 (10)
H1A—C1—H1C	109.5	O4—Dy1—N4	121.11 (11)
H1B—C1—H1C	109.5	O3—Dy1—N4	74.17 (11)
C3—C2—O1	122.0 (4)	O2—Dy1—O6	108.45 (10)
C3—C2—C7	123.2 (4)	O2^i^—Dy1—O6	116.45 (9)
O1—C2—C7	114.8 (3)	O1^i^—Dy1—O6	76.95 (10)
C2—C3—C4	120.1 (4)	O4—Dy1—O6	141.79 (10)
C2—C3—H3	120.0	O3—Dy1—O6	139.92 (10)
C4—C3—H3	120.0	N4—Dy1—O6	69.16 (10)
C5—C4—C3	118.5 (4)	O2—Dy1—N5	139.17 (10)
C5—C4—H4	120.8	O2^i^—Dy1—N5	144.17 (10)
C3—C4—H4	120.8	O1^i^—Dy1—N5	81.46 (10)
C4—C5—C6	122.6 (4)	O4—Dy1—N5	79.22 (10)
C4—C5—H5	118.7	O3—Dy1—N5	79.08 (10)
C6—C5—H5	118.7	N4—Dy1—N5	66.52 (11)
C5—C6—C7	119.6 (4)	O6—Dy1—N5	72.05 (10)
C5—C6—C8	115.0 (4)	O2—Dy1—O8	77.32 (10)
C7—C6—C8	125.3 (4)	O2^i^—Dy1—O8	70.22 (9)
O2—C7—C2	119.8 (3)	O1^i^—Dy1—O8	76.42 (10)
O2—C7—C6	124.3 (3)	O4—Dy1—O8	140.65 (10)
C2—C7—C6	115.8 (3)	O3—Dy1—O8	153.86 (10)
N4—C8—C6	126.1 (4)	N4—Dy1—O8	98.23 (11)
N4—C8—H8	117.0	O6—Dy1—O8	50.52 (9)
C6—C8—H8	117.0	N5—Dy1—O8	121.51 (10)
N5—C9—N9	123.9 (4)	O2—Dy1—Dy1^i^	37.86 (6)
N5—C9—S1	117.3 (3)	O2^i^—Dy1—Dy1^i^	36.10 (6)
N9—C9—S1	118.8 (3)	O1^i^—Dy1—Dy1^i^	103.10 (6)
C11—C10—C15	119.0 (4)	O4—Dy1—Dy1^i^	92.27 (7)
C11—C10—N5	125.8 (3)	O3—Dy1—Dy1^i^	90.23 (7)
C15—C10—N5	115.2 (4)	N4—Dy1—Dy1^i^	113.24 (8)
C12—C11—C10	118.9 (4)	O6—Dy1—Dy1^i^	118.59 (7)
C12—C11—H11	120.6	N5—Dy1—Dy1^i^	169.00 (8)
C10—C11—H11	120.6	O8—Dy1—Dy1^i^	69.47 (6)
C11—C12—C13	120.9 (4)	C8—N4—N9	114.2 (3)
C11—C12—H12	119.5	C8—N4—Dy1	131.6 (3)
C13—C12—H12	119.5	N9—N4—Dy1	114.1 (2)
C14—C13—C12	121.5 (4)	C9—N5—C10	109.1 (3)
C14—C13—H13	119.3	C9—N5—Dy1	113.1 (3)
C12—C13—H13	119.3	C10—N5—Dy1	136.6 (2)
C13—C14—C15	117.4 (4)	O5—N6—O3	122.4 (4)
C13—C14—H14	121.3	O5—N6—O4	122.6 (4)
C15—C14—H14	121.3	O3—N6—O4	115.0 (3)
C14—C15—C10	122.3 (4)	O7—N7—O6	122.5 (4)
C14—C15—S1	127.6 (3)	O7—N7—O8	122.2 (4)
C10—C15—S1	109.9 (3)	O6—N7—O8	115.2 (3)
O9—C16—H16A	109.5	C9—N9—N4	117.0 (3)
O9—C16—H16C	109.5	C9—N9—H9	121.5
H16A—C16—H16C	109.5	N4—N9—H9	121.5
O9—C16—H16B	109.5	C2—O1—C1	114.9 (3)
H16A—C16—H16B	109.5	C2—O1—Dy1^i^	116.7 (2)
H16C—C16—H16B	109.5	C1—O1—Dy1^i^	127.7 (2)
O2—Dy1—O2^i^	73.96 (10)	C7—O2—Dy1	136.2 (2)
O2—Dy1—O1^i^	139.27 (9)	C7—O2—Dy1^i^	117.7 (2)
O2^i^—Dy1—O1^i^	68.12 (9)	Dy1—O2—Dy1^i^	106.04 (10)
O2—Dy1—O4	109.76 (9)	N6—O3—Dy1	95.9 (2)
O2^i^—Dy1—O4	74.78 (9)	N6—O4—Dy1	96.3 (2)
O1^i^—Dy1—O4	74.29 (10)	N7—O6—Dy1	98.6 (2)
O2—Dy1—O3	76.56 (10)	N7—O8—Dy1	95.6 (2)
O2^i^—Dy1—O3	103.27 (9)	C16—O9—H9A	109.5
O1^i^—Dy1—O3	125.83 (10)	C9—S1—C15	88.36 (19)
			
O1—C2—C3—C4	178.1 (4)	C8—N4—N9—C9	162.8 (4)
C7—C2—C3—C4	−2.5 (7)	Dy1—N4—N9—C9	−20.4 (4)
C2—C3—C4—C5	−1.7 (6)	C3—C2—O1—C1	−25.7 (5)
C3—C4—C5—C6	2.8 (7)	C7—C2—O1—C1	154.9 (4)
C4—C5—C6—C7	0.2 (6)	C3—C2—O1—Dy1^i^	162.7 (3)
C4—C5—C6—C8	−179.3 (4)	C7—C2—O1—Dy1^i^	−16.7 (4)
C3—C2—C7—O2	−173.5 (4)	C2—C7—O2—Dy1	−167.5 (3)
O1—C2—C7—O2	6.0 (5)	C6—C7—O2—Dy1	13.8 (6)
C3—C2—C7—C6	5.3 (6)	C2—C7—O2—Dy1^i^	7.9 (5)
O1—C2—C7—C6	−175.2 (3)	C6—C7—O2—Dy1^i^	−170.8 (3)
C5—C6—C7—O2	174.7 (4)	O2^i^—Dy1—O2—C7	175.7 (4)
C8—C6—C7—O2	−6.0 (6)	O1^i^—Dy1—O2—C7	−162.4 (3)
C5—C6—C7—C2	−4.1 (6)	O4—Dy1—O2—C7	108.9 (3)
C8—C6—C7—C2	175.3 (4)	O3—Dy1—O2—C7	67.4 (3)
C5—C6—C8—N4	176.4 (4)	N4—Dy1—O2—C7	−9.4 (3)
C7—C6—C8—N4	−3.0 (7)	O6—Dy1—O2—C7	−71.2 (3)
C15—C10—C11—C12	−1.1 (6)	N5—Dy1—O2—C7	12.5 (4)
N5—C10—C11—C12	−176.9 (4)	O8—Dy1—O2—C7	−111.5 (3)
C10—C11—C12—C13	1.8 (6)	Dy1^i^—Dy1—O2—C7	175.7 (4)
C11—C12—C13—C14	−1.0 (7)	O2^i^—Dy1—O2—Dy1^i^	0.0
C12—C13—C14—C15	−0.5 (6)	O1^i^—Dy1—O2—Dy1^i^	21.83 (19)
C13—C14—C15—C10	1.2 (6)	O4—Dy1—O2—Dy1^i^	−66.82 (12)
C13—C14—C15—S1	176.6 (3)	O3—Dy1—O2—Dy1^i^	−108.32 (12)
C11—C10—C15—C14	−0.5 (6)	N4—Dy1—O2—Dy1^i^	174.87 (13)
N5—C10—C15—C14	175.8 (4)	O6—Dy1—O2—Dy1^i^	113.12 (11)
C11—C10—C15—S1	−176.5 (3)	N5—Dy1—O2—Dy1^i^	−163.24 (12)
N5—C10—C15—S1	−0.2 (4)	O8—Dy1—O2—Dy1^i^	72.77 (11)
C6—C8—N4—N9	−179.2 (4)	O5—N6—O3—Dy1	−175.9 (4)
C6—C8—N4—Dy1	4.8 (7)	O4—N6—O3—Dy1	3.2 (3)
O2—Dy1—N4—C8	0.1 (4)	O2—Dy1—O3—N6	126.4 (2)
O2^i^—Dy1—N4—C8	9.7 (5)	O2^i^—Dy1—O3—N6	56.8 (2)
O1^i^—Dy1—N4—C8	153.4 (3)	O1^i^—Dy1—O3—N6	−15.6 (2)
O4—Dy1—N4—C8	−104.5 (4)	O4—Dy1—O3—N6	−1.92 (19)
O3—Dy1—N4—C8	−79.8 (4)	N4—Dy1—O3—N6	−155.1 (2)
O6—Dy1—N4—C8	116.7 (4)	O6—Dy1—O3—N6	−130.8 (2)
N5—Dy1—N4—C8	−164.5 (4)	N5—Dy1—O3—N6	−86.6 (2)
O8—Dy1—N4—C8	74.6 (4)	O8—Dy1—O3—N6	128.8 (3)
Dy1^i^—Dy1—N4—C8	3.5 (4)	Dy1^i^—Dy1—O3—N6	90.8 (2)
O2—Dy1—N4—N9	−176.0 (3)	O5—N6—O4—Dy1	175.9 (4)
O2^i^—Dy1—N4—N9	−166.4 (2)	O3—N6—O4—Dy1	−3.2 (3)
O1^i^—Dy1—N4—N9	−22.7 (3)	O2—Dy1—O4—N6	−52.3 (2)
O4—Dy1—N4—N9	79.4 (3)	O2^i^—Dy1—O4—N6	−118.6 (2)
O3—Dy1—N4—N9	104.2 (3)	O1^i^—Dy1—O4—N6	170.4 (2)
O6—Dy1—N4—N9	−59.4 (3)	O3—Dy1—O4—N6	1.91 (19)
N5—Dy1—N4—N9	19.4 (2)	N4—Dy1—O4—N6	32.4 (2)
O8—Dy1—N4—N9	−101.4 (3)	O6—Dy1—O4—N6	127.8 (2)
Dy1^i^—Dy1—N4—N9	−172.6 (2)	N5—Dy1—O4—N6	86.3 (2)
N9—C9—N5—C10	−175.2 (4)	O8—Dy1—O4—N6	−146.3 (2)
S1—C9—N5—C10	5.1 (4)	Dy1^i^—Dy1—O4—N6	−86.6 (2)
N9—C9—N5—Dy1	15.4 (5)	O7—N7—O6—Dy1	−176.6 (4)
S1—C9—N5—Dy1	−164.29 (19)	O8—N7—O6—Dy1	3.1 (4)
C11—C10—N5—C9	173.0 (4)	O2—Dy1—O6—N7	−56.8 (2)
C15—C10—N5—C9	−3.0 (5)	O2^i^—Dy1—O6—N7	24.1 (3)
C11—C10—N5—Dy1	−21.3 (6)	O1^i^—Dy1—O6—N7	81.2 (2)
C15—C10—N5—Dy1	162.7 (3)	O4—Dy1—O6—N7	123.1 (2)
O2—Dy1—N5—C9	−40.9 (3)	O3—Dy1—O6—N7	−147.7 (2)
O2^i^—Dy1—N5—C9	167.3 (2)	N4—Dy1—O6—N7	−122.6 (2)
O1^i^—Dy1—N5—C9	135.7 (3)	N5—Dy1—O6—N7	166.3 (3)
O4—Dy1—N5—C9	−148.7 (3)	O8—Dy1—O6—N7	−1.8 (2)
O3—Dy1—N5—C9	−95.1 (3)	Dy1^i^—Dy1—O6—N7	−16.8 (2)
N4—Dy1—N5—C9	−17.7 (3)	O7—N7—O8—Dy1	176.7 (4)
O6—Dy1—N5—C9	56.8 (3)	O6—N7—O8—Dy1	−3.0 (4)
O8—Dy1—N5—C9	67.5 (3)	O2—Dy1—O8—N7	129.1 (2)
Dy1^i^—Dy1—N5—C9	−108.9 (4)	O2^i^—Dy1—O8—N7	−153.6 (3)
O2—Dy1—N5—C10	153.8 (3)	O1^i^—Dy1—O8—N7	−82.3 (2)
O2^i^—Dy1—N5—C10	2.0 (5)	O4—Dy1—O8—N7	−125.1 (2)
O1^i^—Dy1—N5—C10	−29.6 (4)	O3—Dy1—O8—N7	126.7 (3)
O4—Dy1—N5—C10	46.0 (4)	N4—Dy1—O8—N7	56.0 (2)
O3—Dy1—N5—C10	99.6 (4)	O6—Dy1—O8—N7	1.8 (2)
N4—Dy1—N5—C10	177.0 (4)	N5—Dy1—O8—N7	−11.4 (3)
O6—Dy1—N5—C10	−108.5 (4)	Dy1^i^—Dy1—O8—N7	167.8 (3)
O8—Dy1—N5—C10	−97.8 (4)	N5—C9—S1—C15	−4.6 (3)
Dy1^i^—Dy1—N5—C10	85.8 (5)	N9—C9—S1—C15	175.7 (4)
N5—C9—N9—N4	3.2 (6)	C14—C15—S1—C9	−173.3 (4)
S1—C9—N9—N4	−177.1 (3)	C10—C15—S1—C9	2.4 (3)

Symmetry codes: (i) −*x*+1, −*y*, −*z*+2.

### Hydrogen-bond geometry (Å, °)

**Table:**

*D*—H···*A*	*D*—H	H···*A*	*D*···*A*	*D*—H···*A*
O9—H9A···O3^ii^	0.84	2.64	3.206 (4)	126
O9—H9A···O8^iii^	0.84	2.24	3.049 (4)	161
N9—H9···O9^iv^	0.88	1.91	2.751 (4)	160

Symmetry codes: (ii) *x*, *y*, *z*−1; (iii) −*x*+1, −*y*, −*z*+1; (iv) −*x*, −*y*, −*z*+1.
